# Ethics and Medico Legal Aspects of “Not for Resuscitation”

**DOI:** 10.4103/0973-1075.68404

**Published:** 2010

**Authors:** Naveen Sulakshan Salins, Sachin Gopalakrishna Pai, MS Vidyasagar, Manikkath Sobhana

**Affiliations:** Palliative Medicine Consultant, Palliative Medicine Unit, Departments of Radiotherapy and Oncology, Shiridi SaiBaba Cancer Hospital and Research Centre, KMC Manipal, Manipal University 576104, India; 1 Assistant Professor, Department of Medical Oncology, Shiridi SaiBaba Cancer Hospital and Research Centre, KMC Manipal, Manipal University 576104, India; 2 Professor, Department of Radiotherapy and Oncology, Shiridi SaiBaba Cancer Hospital and Research Centre, KMC Manipal, Manipal University 576104, India; 3 Medical Social Worker, Department of Radiotherapy, and Oncology, Shiridi SaiBaba Cancer Hospital and Research Centre, KMC Manipal, Manipal University 576104, India

**Keywords:** Autonomy, Cardiopulmonary resuscitation, Ethics, Law

## Abstract

Not for resuscitation in India still remains an abstract concept with no clear guidelines or legal frame work. Cardiopulmonary resuscitation is a complex medical intervention which is often used inappropriately in hospitalized patients and usually guided by medical decision making rather than patient-directed choices. Patient autonomy still remains a weak concept and relatives are expected to make this big decision in a short time and at a time of great emotional distress. This article outlines concepts around ethics and medico legal aspects of not for resuscitation, especially in Indian setting.

Life sustaining treatment is defined as any medical intervention, technology, procedure or medication that is administered to a patient in order to forestall the moment of death, whether or not the treatment is intended to affect the underlying life threatening disease or biological process. Decisions to withhold life-sustaining treatment are made in two different situations. In the first, treatment is withheld from an actively dying person whose existing condition indicates that effective cardiopulmonary resuscitation (CPR) is unlikely to be successful or a successful CPR is likely to be followed by a length and quality of life that would not be in best interests of patient to sustain. In the second, the decision is hypothetical, whereby the withholding of treatment is made in advance, in a situation where a life threatening condition may eventuate.[1]

Cardiopulmonary resuscitation (CPR) came into widespread use in 1960s and soon it was apparent that it was inappropriately used in some patients most obviously in advanced metastatic malignancy, end stage organ failure and severe sepsis. CPR is a form of intensive and invasive treatment associated with high mortality. Compared to other treatments, this intensive treatment is poorly discussed and documented.[2]

When a patient suffers sudden cardiopulmonary arrest usually, the decision whether or not to resuscitate depends upon the physician’s professional appraisal of the likelihood of successfully restoring cardiopulmonary functioning of a particular patient versus the probable futility of a resuscitative attempt. However, ethical, legal and sometimes financial implications must be taken into account. The issue of resuscitation raises fundamental ethical questions about autonomy (patient’s wishes and choices), beneficence (appropriate decision making), non -maleficence (harm avoidance) and justice (allocation of limited resources). Medico legal aspects of CPR deal with issues such as competency of an individual in decision-making, standards and processes of decision-making and dilemmas in instituting or withholding CPR in an incompetent individual.[3]

In the western world, more than 90% of patients reported to have information about CPR from television. Patients often overestimated their likelihood of survival after CPR and this misinformation often led to choosing resuscitation in situations in which successful outcomes were extremely unlikely.[4,5] A study of popular medical dramas projected an unusually high survival rate following a CPR in contrary to the actual figures.[6] Often patients and patient’s relative’s participate in decisions about resuscitation and end of life at a time of great emotional distress. In a short period of time, they are expected to digest and evaluate complex medical information and make decisions about themselves or for the loved ones. Therefore, prior knowledge, information and media portrayal strongly influence the decision making.[7] A study reported that 50% of elderly patients, who first chose to be resuscitated, changed their opinion after they received more detailed information about the CPR process and possibility of surviving.[5] Educational intervention consisting of handouts and a videotape about advance directives improved knowledge and intended to change attitudes and behavior about resuscitation.[8] It is important for the physicians to explain the process, clinical accompaniments and aftermath including intubation, mechanical ventilation, artificial feeding, hydration, supplemental oxygen and pharmacological agents. Therefore, decision of instituting CPR is not a single ethical decision but a number of choices either bundled together or spread over a period of time.[9]

In the past, issuing a ‘not for resuscitation’ order was considered as a part of doctor’s ‘therapeutic prerogative’ and was often not formally registered. This practice still exists in many places and countries. The main ethical problems with such informal practices are that they exclude patients and relatives from the process of decision-making process and give paternalistic doctors absolute power over the patient.[10] A Hungarian study showed that medical practice in that country is rather paternalistic and the most important factor influencing the decision of ‘not for resuscitation’ was the opinion of the doctor in charge.[11] Indian medical practice works on similar lines. Resuscitation status should not be just based on medical grounds and value input from patients and families should always be considered. Successful outcomes of resuscitation mean restoration of patient’s health to their pre-arrest state. Hence, resuscitation must be instituted in only those patients where there is a reasonable chance of restoring cardiopulmonary functions, optimal mental capacity and length and quality of life that would be in the best interests of patients to sustain.[12] CPR is considered futile if its purpose cannot achieve reasonable length and quality of life and qualitative definition of futility must include low chance of survival and low quality of life afterward. The framework for decisions about futility can be best achieved by accurate prognostication, good communication, respecting patient choices, value opinion of other healthcare providers, local society factors, and cultural and social consensus.[13] In 2005, The Royal College of Anesthetics, Physicians, Intensive Care Society and Resuscitation Council (UK) published new resuscitation standards. The recommendations made it mandatory: (a) to identify patients in whom cardiopulmonary arrest is an anticipated terminal event and institution of CPR is inappropriate (b) for all institutions should ensure that there is a clear and explicit resuscitation plan for all patients (c) when there is no resuscitation plans and wishes of the patient are unknown, the resuscitation decision should be made by the attending medical emergency team in the best interests of the patient.[14]

Patient autonomy should be cornerstone in deciding about patient’s resuscitation status. Accurate information about the condition, prognosis, and nature of the proposed intervention, alternatives, risks and benefits may enable the patients to make better decisions about resuscitation and end of life. Physicians seldom discuss advance directives even with their seriously ill patients.[15] Advance directives are the term applied to any expression of a person’s thoughts, wishes, or preferences for his or her end of life care. It usually specifies nature and extent of care and it could be based on conversations, written directives, and living will or through medical power of attorney. In a living will, the patient gives directions to physicians about provision of medical care should the patient become terminally ill and unable to make decisions. Competent patients in anticipation of future incompetence could appoint a family member or friend as a surrogate decision maker and grant durable power of attorney that allows them to take medical decisions for the patient if he or she loses the capacity to make their decisions. Surrogate decision makers should always base their decisions on patient’s previously expressed wishes. Physicians can always override the decisions of the surrogate decision makers if these decisions are not made in the best interests of the patient. It is important to explore the opinions of surrogate decision makers and provide them knowledge and information to make the best possible decision. If there are non-negotiable difference of opinion between doctors and surrogate decision makers regarding appropriateness of resuscitation, then it should be further explored and should always be referred to the hospital ethics committee.[16,17] Decision making by doctors regarding resuscitation and end of life care depends upon patient-dependent factors and personal characteristics of doctor. Doctors should be open to discuss end of life preferences openly with other health care providers and surrogate decision makers. Junior doctors in training would benefit from the supervision of the experienced colleagues in dealing with process of end of life care and ‘not for resuscitation’.[18,19]

The first hospital policies on ‘not for resuscitation’ were published in medical literature in 1976. Results from the MERIT study showed that in UK 22% of patients attended by a medical emergency team (MET) team are issued ‘not for resuscitation’ order at the time or after a MET call and in Australia 23% of all MET calls were appropriate for ‘not for resuscitation’ order and 3.8% of the patients were issued ‘not for resuscitation’ order during the time of medical emergency team attendance.[20] Dutch hospital study showed medical staff were poorly documenting ‘not for resuscitation’ orders. The possible barriers for limited documentation were: (a) inability to discuss resuscitation decisions (b) lack of knowledge about the facts and consequences of resuscitation and (c) unwillingness to make resuscitation open for discussion.[21]

An American hospital study identified similar barriers for not documenting ‘not for resuscitation’ and in addition other barriers were: (d) not expecting the patient to die during admission (e) waiting for the patient’s own doctor (f) not having enough knowledge about a particular patient (g) not finding the right moment or spot to discuss it and (h) just forgetting about it.[22]

In India there is no formal process of discussion and documentation of ‘not for resuscitation’. Patient autonomy still remains a weak concept and surrogate decision making by the next of kin or the financial provider usually overrides patient wishes.[23] Up to 80% of the health care bills are paid by the patients and less than 20% depend on the public health care, which is severely crippled with acute bed shortages and lack of infrastructure. Socio-economic considerations complicate patient autonomy, issues around resuscitation and delivery of end of life care.[24] The Indian Society of Critical Care Medicine in 2005 proposed guidelines for limiting life-prolonging interventions and providing palliative care at end of life. The doctors are morally and ethically obliged to provide good prognostication and initiate discussions around treatment options, benefits of life prolonging treatment, and resuscitation. Patients and relatives should be well informed about realistic outcomes of a disease modifying treatment, withholding and withdrawing treatment.[25] In 2002, 122 Indian physicians participated in a study that evaluated “physician’s beliefs regarding end of life care”. Majority of physicians did not apply ‘not for resuscitation’ order and in most of the cases applied these were primarily discussed with the family. In 2008, AIIMS study, 40 Indian pediatricians participated in end of life survey and the 3 major factors influencing withholding support in a critically ill child was a) child’s likelihood of survival b) potential for neurologically intact survival and c) quality of life. The results of Indian pediatrician’s end of life practice survey showed that 45% of pediatricians initiated “not for resuscitation” orders in a dying child. 55% of them withheld active treatment and none had withdrawn active treatment. Prognosis and limiting life prolonging measures were discussed with the families. Other clinical units and supervisors were consulted during discussion about ethics of “not for resuscitation”. The study of deaths in Indian ICU setting showed only 22% had life limiting management when compared to 74% in the western setting. Among the 298 deaths only 4 had “Not for resuscitation” orders.

Left against medical advice (LAMA) is a common situation seen in Indian setting where family unilaterally withdraws treatment due to financial constraints.

Indian Society of Critical Care Medicine in 2005 put forth a position paper outlining guidelines for “not for resuscitation” in an Indian setting. The recommendations were a) Duty of the physician to discuss with honest and clarity regarding prognosis and treatment options b) When the fully informed capable patient or family desires to consider palliative care, the physician should offer the available modalities of limiting life-prolonging interventions c) Physician must discuss the implications of forgoing aggressive interventions through formal conferences with the capable patient or family, and work toward a shared decision-making process d) If there is a conflict then pending consensus all active treatment should continue e) Responsibility, initiation and implementation of decision of “not for resuscitation” rests with the treating physician f) Clear documentation of the decision, directives and end of life wishes h) Withdrawal of life support should be consistent with good practice, ethically right and within the limits of existing law i) Physician is obliged to provide compassionate and effective palliative care to the patient and family. The absence of guidelines for withdrawal and withholding of life support in Indian law is perceived to be the most important obstacle for providing good end of life care. The study found that the barriers for providing good end of life care were primarily legal, administrative and lack of hospital policies and were not due to ethical and cultural barriers.[26] Indian legal system maintains an ambiguous stance towards issues relating to ‘limiting life support’, ‘withholding and withdrawing treatment’ and ‘resuscitation’. The Supreme Court of India in 1994 ruled that an attempt to hasten death in terminal illness might be viewed as a natural process. “A person cannot be forced to enjoy the right of life to his detriment, disadvantage or dislike”.[27] The above judgment of the Supreme court was overruled by the Constitution bench which ruled that permitting termination of life in the dying or vegetative state is not compatible with Article 21 of the Constitution which states that “No person shall be deprived of his life or personal liberty except according to procedure established by law”.[27] The interpretation of Gian Kaur case disallows the concept of Euthanasia as it violates Article 21 of the Indian Constitution. As withholding and withdrawing life support amounts to abetment of suicide and abetment of suicide is a punishable offence according to Indian Penal Code. These issues are addressed by the Law commission of India and until such laws come into effect patient autonomy, family wishes and medical decision making at end of life will still remain guided by the Indian Penal Code.[24]
